# Mechanisms Mediating Nuclear Trafficking Involved in Viral Propagation by DNA Viruses

**DOI:** 10.3390/v11111035

**Published:** 2019-11-07

**Authors:** Guohui Li, Xinyu Qi, Zhaoyang Hu, Qi Tang

**Affiliations:** Institute of Life Sciences, Jiangsu University, Zhenjiang 212013, Jiangsu, China; ghli@ujs.edu.cn (G.L.); sunnyhu163@163.com (Z.H.)

**Keywords:** viral propagation, NLSs, cNLSs, ncNLSs, viral replication, expression regulation, virion assembly

## Abstract

Typical viral propagation involves sequential viral entry, uncoating, replication, gene transcription and protein synthesis, and virion assembly and release. Some viral proteins must be transported into host nucleus to facilitate viral propagation, which is essential for the production of mature virions. During the transport process, nuclear localization signals (NLSs) play an important role in guiding target proteins into nucleus through the nuclear pore. To date, some classical nuclear localization signals (cNLSs) and non-classical NLSs (ncNLSs) have been identified in a number of viral proteins. These proteins are involved in viral replication, expression regulation of viral genes and virion assembly. Moreover, other proteins are transported into nucleus with unknown mechanisms. This review highlights our current knowledge about the nuclear trafficking of cellular proteins associated with viral propagation.

## 1. Introduction

**Figure 1:**
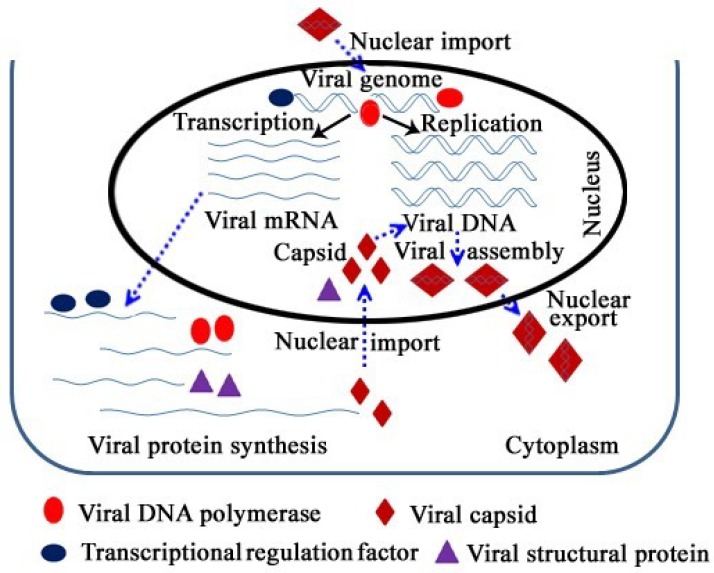
Schematic representation of the nucleocytoplasmic shuttling of viral proteins and particles.

Nuclear trafficking of cellular proteins is critical for the regulation of cellular processes, including DNA replication, transcriptional regulation, gene expression, signal transduction and apoptosis [1]. However, the double-membraned nuclear envelope restricts macromolecular movement between the cytoplasmic and nuclear compartments in eukaryotic cells [2]. Some proteins are required to execute their accurate and specific cellular functions at specific times and in the correct locations within cells. Therefore, dynamic nucleocytoplasmic shuttling plays an important role in the regulation of cellular function, and the appropriate proteins need to be translocated and further activated in the right place via control of nuclear import and export. A prominent example regulated in this way is viral propagation, as viral biological processes occur in a specified cellular compartment [3,4]. For instance, the series of events in viral infection, such as viral replication, transcription and assembly, usually occur in the cell nucleus [5], while translation, post-translational modification and protein degradation usually occur in the cytoplasm [6,7,8]. Most DNA virus replication and capsid assembly steps take place in the nucleus of infected cell. However, the mechanisms underlying such processes as the delivery of viral proteins to the nucleus and the export of progeny virus from the nucleus remain elusive. It is evident that nuclear transport signals play an important role in these processes. To their own advantage, the recruitment of some cellular cofactors is required to ensure the occurrence of cellular events during viral propagation. Therefore, intracellular trafficking between nucleus and cytoplasm—including that of the viral capsid, viral genome, viral polymerase, and some transcriptional regulation factors—is a prerequisite for viral propagation and modulation of the interactions between hosts and viruses. Some key shuttling events involved in the infection of a host by DNA viruses are shown schematically in Figure 1, below.

Nuclear import of numerous proteins is mainly mediated by nuclear localization signals (NLSs), and these trafficking events are essential for expression regulation of functional proteins in biological processes, such as cell-cycle progression, signal transduction, viral infection and the replication cycle of diverse viruses [9,10,11,12]. Nucleocytoplasmic trafficking of functional proteins across the nuclear membrane occurs through the nuclear pore complex (NPC), which has been exploited to develop new diagnostic and therapeutic targets [13,14]. It is generally accepted that some small proteins (<40 kDa) can passively diffuse between cytoplasm and nucleus, and NLSs are required for nuclear import of larger proteins [15,16]. NLSs are characterized by clusters of basic amino acids, which can be classified into classical (cNLS) and non-classical (ncNLS) types. The cNLSs are further divided into classical monopartite NLSs and bipartite NLSs. In this paper, we summarize the current knowledge about nuclear import of proteins involved in viral propagation, which may help us to better understand the molecular pathogenesis of viruses.

## 2. Diversity of NLSs Identified in Viral Proteins

A growing number of viral proteins are known to be transported into the nucleus of host cells. Monopartite NLSs were first identified in the SV40 large T antigen, which contains a continuous stretch of basic amino acids [17]. Bipartite NLSs contain two clusters of basic residues separated by a short amino acid linker, which is regarded as a potential NLS [18]. Some unconventional NLSs have been identified in the human parvovirus B19 major capsid protein (VP2) and parvovirus minute virus of mice VP2, which contain some basic amino acids (K and R) in KLGPRKATGRW and KGKLTMRAKLR [19,20]. Surprisingly, the coiled-coil domain of signal transducer and activator of transcription (STAT5) functions as an unconventional NLS [21], which does not conform to a conventional monopartite or bipartite NLS.

**Figure 2:**
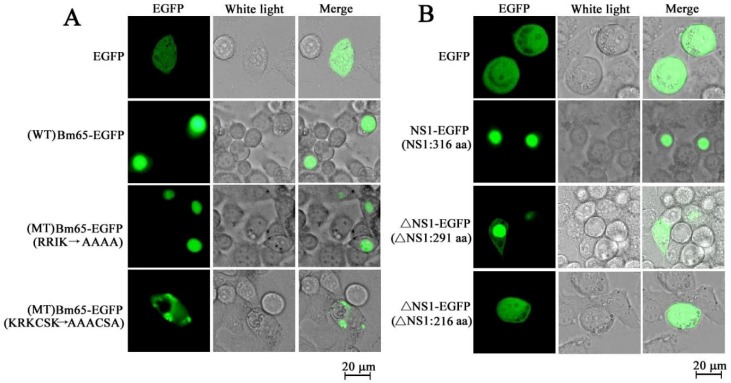
Distribution of green fluorescence signal in BmN cells transfected with a transient expression vector. (**A**) Effect of mutations in the motifs of ^33^RRIK and ^76^KRKCSK of Bm65 on the subcellular localization of the Bm65–EGFP fusion protein. Compared with the wild type (WT) and the mutation in ^33^RRIK of Bm65, the mutations in the motif of ^76^KRKCSK block the nuclear import of Bm65. (**B**) Effect of N-deletion of BmBDV NS1 on subcellular localization of the NS1–EGFP fusion protein. Compared with NS1–EGFP, deletions of the N-terminal of BmBDV NS1 impaired the nuclear import of NS1. The WT indicates the amino acid sequence of Bm65 WT. EGFP indicates enhanced green fluorescence protein. The amino acids of RRIK were mutated into ^33^AAAA, and ^76^KRKCSK into ^76^AAACSA in the mutant type (MT) of Bm65. WT BmBDV NS1 consists of 316 amino acids (aa), with 291 aa and 216 aa indicating the deletion of 25 and 100 amino acids from the N-terminal of BmBDV NS1, respectively.

Some baculovirus proteins, such as polyhedron, 38K, VP39, Bm65 and LEF-11, have also been reported to exert their functions in the nucleus [22,23,24,25,26]. Researches have further revealed some cNLSs in their amino acid sequences. For example, the KRKK sequence has been identified as necessary for the nuclear localization of polyhedron [22], whereas R(537) and R(538) of IE1 function as a dimeric nuclear localization element for nuclear import and promoter activation [27]. Bm65 is an early protein associated with the repair of ultraviolet-induced DNA damage, and accumulates mainly in the nuclei of infected cells [24,26,28]. Li et al. (2019) have revealed the mechanism of Bm65 importation into nucleus through analysis of transient expression and a series of mutations (unpublished). The study’s transient expression assay indicated that the ^76^KRKCSK motif was necessary for the nuclear localization of Bm65, but ^33^RRIK had no effect on Bm65’s nuclear import (Figure 2A). Additionally, BmBDV NS1 was found to be a multifunctional protein that accumulated mainly in nucleus to perform its function. The phosphorylation sites of BmBDV NS1 were characterized and the phosphorylation was reported to regulate the activity [29,30]. Li et al. [30] further demonstrated that the 100 N-terminal amino acids of BmBDV NS1 have an important role in controlling the transport of BmBDV NS1 from cytoplasm to nucleus (Figure 2B). The NLS of Bm65 conforms to the structure of a cNLS, and the N-terminal domain of BmBDV NS1 potentially functions as an ncNLS. Mutations in these NLSs result in failure of nuclear import of target proteins, which is likely to impair or inhibit the generation of mature virions. 

From the above description of NLSs identified in viral proteins, it is believed that the observed diversity of NLSs inevitably arose from various different sequences and the complexity of amino acid composition. The identification of diverse NLSs may provide important information for the development of new anti-viral drugs. 

## 3. Entry of the Viral Genome and Capsid

### 3.1. Nuclear Import of the Viral Genome

Most DNA viruses replicate their genomes in the nucleus of an infected cell. According to the general concept of viral proliferation, the replication of most DNA viruses requires delivery of the viral genome into the host cell’s nucleus. In this way, viruses are able to replicate their genomes in the nuclei of infected cells in the early stages of viral infection, while the synthesized viral genome and capsids are assembled into new viral particles during the latter stages. However, the viral genome is too large to passively diffuse through the NPC, and so it must be actively transported into the nucleus. As a result, there are questions concerning the entry of the viral genome into the host cell’s nucleus and the subsequent release of assembled viruses into the cytoplasm. Nuclear targeting of proteins depends on the recognition of a stretch of basic amino acids by cellular transport proteins. However, the nuclear delivery mechanism of the viral genome has not been fully elucidated. 

Many viruses depend on nuclear proteins for replication. Therefore, the viral genome is required to enter the nucleus of a host cell for viral DNA synthesis. Upon reaching the cellular compartment where viral replication occurs, the viral genome is released from the capsid or nucleoprotein complex. To date, viruses have evolved to exploit diverse strategies for the nuclear import of viral nucleocapsids. Taking baculoviruses as an example, several modes have been proposed for the nuclear import of their nucleocapsids. In agreement with the replication of most DNA viruses, the rod-shaped, 250-nm long nucleocapsids of baculoviruses must deliver their genomes into the host cell’s nucleus for replication. Nucleocapsids from baculoviruses in the genus granulovirus (GV) can eject their nucleic acids into the nucleus by docking at the cytoplasmic side of an infected cell’s nuclear pore complex (NPC), leaving the empty capsids at the NPC [31]. Recently, Au et al. [32] reported a new mechanism for nuclear import by a baculovirus nucleocapsid, which utilized actin-based propulsion. In the proposed model, Autographa californica multicapsid nucleopolyhedrovirus (AcMNPV) exploits the propulsive force of actin polymerization to drive migration of the nucleocapsid through the cytoplasm, as well as to achieve nuclear import of the nucleocapsid. These contradictory findings suggest that the mechanism of nuclear import by baculovirus nucleocapsids is genus specific. A similar mechanism is now well-established for the genome delivery of herpes simplex virus (HSV). In this mechanism, dynein is used to propel nucleocapsids to the cytoplasmic side of the NPC and the viral genome is injected into the nucleus, leaving an empty capsid at the NPC [33,34]. Moreover, the motility mediated by dynein has been reported to deliver the adenovirus capsid to the NPC. Unlike HSV-1, the viral genome and capsid proteins travel through the NPC into the nucleus [35]. Papillomaviruses are non-enveloped DNA viruses with the major capsid protein L1 and minor capsid protein L2, which can cause malignancies such as cervical cancer [36,37]. Like most DNA viruses, papillomaviruses must deliver their genomes to the host cell’s nucleus to initiate viral transcription and replication. A new model for papillomaviruses genome delivery was proposed by Aydin et al. in 2017 [38]. In the model, a central region in the minor capsid protein L2 was identified to facilitate viral genome tethering and membrane penetration for mitotic nuclear entry, which required initiation of mitosis associated with breakdown of the nuclear envelope. The conventional mechanism for genome delivery is that specific regions of proteins mediate nuclear import of the viral genome by the formation of a protein–DNA complex. Conversely, the NLSs at the N and C termini of papillomavirus L2 are not necessary for the complexes involving the viral genome and L2 through the nuclear pores. 

As far as the hepatitis B virus (HBV) is concerned, there are two possibilities of how the HBV genome enters the nucleus. One possibility is that the specific regions of some nucleocapsids mediate nuclear import of the viral genome. For example, the PreS2 domain of HBV is a membrane-permeable peptide designated the translocation motif (TLM). The TLM is conserved in all hepadnaviridae, and fully assembled nucleocapsids with TLM-peptides on their surface can deliver packaged nucleic acids to the nucleus [39,40]. Another possibility is facilitation of the formation of a polymerase–DNA complex, which mediates nuclear import of the viral genome. For example, the entry of the genome complex into the nucleus can be mediated by HBV polymerase [41,42]. The mechanism of nuclear import of viral genomes definitely exists, but different viruses have already been shown to use different mechanisms. As the size and structure of viruses vary enormously, each virus has evolved a unique strategy to deliver its genome into the nucleus. Evidently, DNA viruses have evolved a wide variety of strategies to invade host nuclei, and take advantage of host cell machinery for DNA replication and viral production. According to the different strategies exploited by DNA viruses, it is believed that NLSs play an important role in targeting the viral capsid to the NPC prior to nuclear entry.

### 3.2. Requirements for Nuclear Transport of Viral Capsids

**Figure 3:**
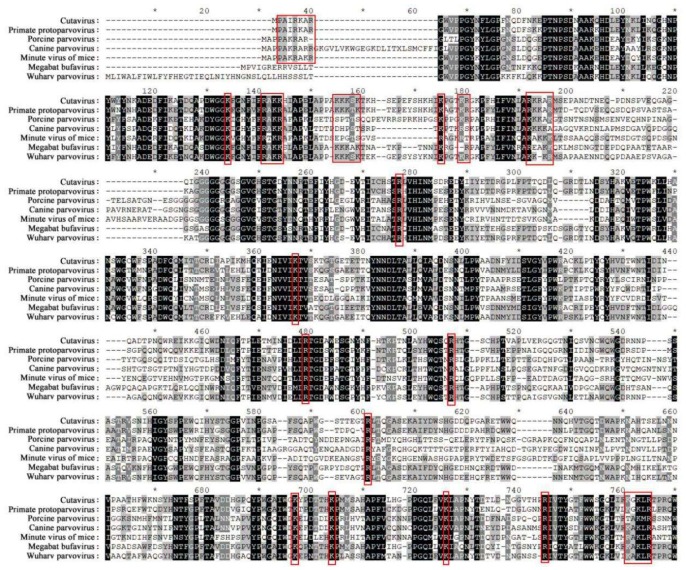
Multiple sequence alignment showed that basic amino acid clusters, corresponding to potential NLSs, are found in viral capsid sequences. Some single amino acids (lysine (R) and arginine (K)) are highly conversed in these sequences, which are potentially involved in nuclear import. These sequences are from GenBank, and their accession numbers are as follows: cutavirus (AMS35095.1), Wuharv parvovirus (AFV48070.1), primate protoparvovirus (YP_009507380.1), megabat bufavirus (BAU69605.1), minute virus of mice (NP_041244.1), canine parvovirus (NP_041400.1) and porcine parvovirus (NP_757371.1). Clusters of basic amino acids and some highly conserved R and K are presented within a red frame.

Parvoviruses are a group of non-enveloped single-stranded (ss) DNA viruses with genomes 4–6 kb in length. They are divided into two groups, *Parvovirinae* and *Densovirinae*, and include the minute virus of mice, porcine parvovirus, and human parvovirus B19 [43,44]. Most of these viruses are characterized by their capsids with nuclear targeting [45]. Alignment studies have revealed that four clusters of basic amino acids and some single amino acids are highly conserved in the sequence of parvovirus capsids (Figure 3). The basic amino acids are regarded as potential NLSs, which may play an important role in directing the nuclear transport of capsids. Some basic amino acids in the capsids have been identified as efficient NLSs for nuclear targeting. For example, the N-terminal residues 4–13 (PAKRARRGYK) of the VP1 capsid have nuclear targeting activity [46], and the basic amino acid cluster of PAKRAKR is a cNLS for nuclear import of porcine parvovirus capsids [47]. A recent study found a highly conserved NLS motif in five viruses of the *Densovirinae* subfamily, which was confirmed to drive trafficking of the newly synthesized Blattella germanica Densovirus (BgDV1) capsid proteins into the host nucleus [48,49]. 

Like parvoviruses, Bombyx mori bidensovirus (BmBDV) is also a small, non-enveloped, linear ssDNA virus, and it was previously classified as a member of the *Parvovirus* genus in *Parvoviridae* [50,51]. However, as the BmBDV genome encodes a protein-primed type B DNA polymerase (pPolB) and contains two viral DNA segments, BmDV-2 is excluded from the family of *Parvoviridae* and is instead classified in the new family *Bidnaviridae* [52,53,54]. However, no cNLSs have been found in BmBDV-encoded proteins, including in the BmBDV capsid [53,55]. At present, the mechanism of nuclear import of the BmBDV VP capsid remains unclear. Research has shown that a transient expression vector is constructed for expression of BmBDV VP-EGFP under the control of an *ie1* promoter. Subsequent fluorescent observations have revealed uniform distribution of green fluorescence in BmN cells. Therefore, further research is required to determine the transport mechanism of BmBDV capsids into host nuclei.

According to the discussion above, it can be inferred that viruses apply different strategies for nuclear import of their viral genomes. The disassembly and uncoating of capsids can occur in either the cytoplasm or nucleus, as some virus capsids stay outside of the nucleus while others enter the nucleus. For example, herpesviruses and adenoviruses attach to the cytoplasmic side of the NPC and eject their genome into the host nucleus, whereas the capsids of HBV and baculoviruses may enter the nucleus intact and therefore disassemble within. As far as parvovirus is concerned, the nucleocapsids do not use the NPC to deliver their genome, but rather disrupt the capsid for nuclear import. In summary, NLSs clearly perform unique functions in these different strategies used by different viruses. Such variation may arise from the size and structure of different DNA viruses, which vary enormously.

## 4. Requirements for the Localization of Viral Self-Encoded DNA Polymerase

Viral replication is contingent on DNA-dependent DNA polymerase. Some viruses can encode DNA polymerase to guide viral DNA synthesis in the nucleus of host cells. For the viruses, the replication and transcription of their viral genome, as well as viral particle assembly, occurs within the nuclei of infected cells, which necessitates the timely nuclear import of viral proteins. To date, most viral self-encoded DNA polymerases have been shown to contain some NLSs, which correctly direct their localization to facilitate viral replication. However, some double-stranded (ds) DNA viruses can replicate in the cytoplasm of host cells, and therefore do not require the nuclear import of DNA polymerase. As a result, no NLSs are found in the sequences of such viral DNA polymerases. Moreover, ssDNA viruses can exploit the host cell’s DNA polymerase to replicate in the nucleus, with the exception of BmBDV. The differences among viral DNA polymerases encoded by DNA viruses are very likely to be a consequence of the evolution of the viral genome.

### 4.1. Replication of Double-Stranded DNA Viruses in the Cytoplasm 

Replication of some DNA virus genomes occurs either at cytoplasmic or nuclear sites. Most nucleocytoplasmic large DNA viruses (NCLDVs), including *Poxviridae*, *Asfarviridae*, *Iridoviridae*, *Ascoviridae*, *Phycodnaviridae*, *Mimiviridae* and *Marseilleviridae* families, replicate and express exclusively in the cytoplasm of infected cells [5,56]. Some NCLDVs, such as vaccinia virus (VACV) and African swine fever virus (ASFV), encode viral polymerase for synthesis of their viral DNA in the cytoplasm [57,58]. For example, VACV is a large, enveloped virus with dsDNA. VACV encodes most of the viral replication machinery including its own DNA polymerase, which is a member of the B family of replicative polymerases encoded by the E9L gene. VACV replication mainly takes place in the cytoplasm, and therefore does not require translocation of VACV DNA polymerase into host cell nuclei [58]. Concordant with the site of VACV replication, the ASFV genome is also replicated and expressed in the cytoplasm. ASFV encodes a DNA polymerase consisting of 174 amino acids which belongs to the polymerase (Pol)X family of DNA polymerases, and is the smallest naturally occurring DNA-directed DNA polymerase described so far [59]. Further research has revealed that ASFV PolX plays an important role in the repair of damaged viral DNA during ASFV infection [60]. DNA polymerases are characterized by their cytoplasmic localization for viral DNA synthesis, and they do not contain NLSs in their sequences. Until now, no progress has been made on this topic. So, NLSs may be involved in the evolution process of viral replication sites.

### 4.2. Replication of dsDNA Viruses in Host Cell Nuclei

Except for the NCLDVs, the replication of other DNA viruses—including double-stranded DNA baculoviruses, herpesviruses and adenoviruses—takes place in the nucleus [61]. Although the method of replication and components of the replisome may vary between different DNA viruses, several fundamental similarities exist among DNA viruses that replicate in the nucleus. These similarities originate from the requirement for viral replication complexes and a specialized nuclear compartment. A key component of viral replication complexes is DNA-dependent DNA polymerase, and most DNA viruses, such as baculoviruses, HBV and herpesviruses, exploit virally self-encoded DNA polymerase to proceed with the synthesis of viral DNA [57,62,63,64,65,66]. As a crucial enzyme in viral replication, DNA polymerase is also the main target for antiviral therapeutics. Some inhibitors against virus-encoded polymerases have been developed for antiviral therapy. In particular, HBV polymerase inhibitors have been widely used in clinical treatment and can maintain viral suppression in patients with chronic hepatitis B [67,68]. 

Viral DNA polymerases are synthesized in the cytoplasm and subsequently translocated into host nuclei for viral DNA replication. Some DNA viruses, including dsDNA and ssDNA viruses, can cause infection and different symptoms in humans. Therefore, the identification of viral DNA polymerase NLSs will be helpful for the development of novel antiviral drugs. Adenovirus DNA polymerase is mediated by bipartite cNLSs [69], while some functional NLSs have been identified in human cytomegalovirus (HCMV) DNA polymerase UL54 and HSV-1 DNA polymerase UL30 [70,71,72]. A novel bipartite cNLS has also been identified in HBV polymerase [34]. These NLSs have an important role in mediating nuclear import of viral DNA polymerases for viral replication. DNA polymerase is highly conserved in all baculoviruses and is crucial in viral DNA replication, and some novel NLSs have been identified in baculovirus-encoded viral DNA polymerases. For example, the DNA polymerase of AcMNPV contains a typical bipartite cNLS motif at residues 804–827 and a monopartite cNLS motif at 939–948. A cNLS has also been identified at the C-terminal residues 827–838 of Spodoptera litura nucleopolyhedrovirus-encoded DNA polymerase [73,74]. Pseudorabies virus (PRV) has a dsDNA molecule 143 kb in length, and viral DNA polymerase is required for viral DNA replication in the nuclei of infected cells. Further research has revealed that PRV UL42 contains a functional and transferable bipartite cNLS at amino acids 354–370 and that K(354), R(355) and K(367) are important for NLS function [75]. Thus, NLSs are essential for nuclear import of viral DNA polymerases, which guide the synthesis of viral DNA in the nucleus, thereby potentially protecting newly synthesized viral DNA from nuclease degradation. 

### 4.3. Replication of ssDNA Viruses in Host Cell Nuclei

Aside from the above dsDNA viruses, viruses with ssDNA genomes that replicate exclusively in the nuclei of infected cells constitute a large class of economically, medically and ecologically important pathogens [76]. BmBDV DNA has two large genome segments, VD1 (6543 nucleotides (nt)) and VD2 (6022 nt), that are packaged into separate capsids, and the virus replicates only in silkworm midgut columnar cells, where it causes a fatal disease [51,53]. Almost all ssDNA viruses lack DNA polymerase genes, but BmBDV is an exception. BmBDV acquired a type B DNA polymerase gene by horizontal gene transfer, and purportedly replicates its genome via a protein-primed mechanism [51,76,77]. However, the mechanism of nuclear import of DNA polymerase encoded by BmBDV is still unclear, which limits our understanding of its dynamic role in infected cells. A greater insight into the functional research of BmBDV polymerase is required to illuminate this scientific issue. 

The *Parvoviridae* family comprises a group of small icosahedral, non-enveloped ssDNA viruses [78]. These viruses occur in both invertebrates and vertebrates, including mammals. Parvoviruses are a group of ssDNA viruses that infect a wide range of animal species and humans. Although not all parvoviruses are pathogenic to humans, some can cause life-threatening human diseases, such as human B19 virus and human bocavirus 1 (HBoV1). The genomes of B19 and HBoV carry limited genetic information, which is usually split into two parts. The left part encodes non-structural (NS) proteins, while the right part encodes two or three structural proteins [79,80,81]. Due to the lack of a DNA polymerase gene in these viral genomes, they utilize host cellular DNA replication machinery for viral DNA replication. Notably, B19 and HBoV are two parvoviruses known to be pathogenic to humans, and can lead to the development of various diseases such as respiratory disease, autoimmune disease and myocarditis [82,83,84,85]. Such afflictions show that B19 and HBoV may have a critical role in the development of pediatric lung disease, as well as immunodeficiency. 

Some NLSs of viral DNA polymerases have been identified and summarized in Table 1. Viral DNA polymerases with some mutations in NLSs fail to accumulate in the nucleus as a result of blocking or impairment of viral DNA replication [73,74,75,86]. Although differences exist in the amino acid sequences of these NLSs, they play an important role in passing through the corresponding NPCs. Collectively, NLSs are usually characterized by one or two clusters of basic amino acids that are required for nuclear import of DNA polymerase, viral DNA synthesis, and virus production.

With reference to the above discussion, it is clear that NLSs are required for nuclear import of viral DNA polymerase. Further, mutations in NLSs in viral DNA polymerases appear to result in failure of viral DNA replication, with the NLSs of other viral proteins also being important for viral replication. As we know, some non-structural proteins, such as LEF-11, large T antigen and E1, are also important for viral replication [25,89,90,91], with studies showing these proteins are mediated into the nucleus by NLSs. The monopartite NLSs of PKKKRKV (residues 126 to 132) were first identified in the SV40 large T antigen [17]. Pan et al. reported a novel NLS in the baculovirus late expression factor 11, and further research revealed that the NLS of baculovirus LEF-11 is important for viral DNA replication [25,88]. It has been reported that the cNLSs of baculovirus IE-1 and ME53 are critical for optimal levels of budded virus production [27,92]. Moreover, Yu and colleagues identified a bipartite NLS of KRK (residues 83 to 85) and KKVK (residues 120 to 123) that is essential for the final destination of E1 in the nucleus to exert its function during viral replication [93]. It is worth mentioning that nuclear accumulation of E1 is regulated by post-translational modifications [94]. In a word, NLSs are necessary for the situating of a number of proteins in the nucleus, directly affecting viral genome replication and viral propagation.

## 5. Mechanism of Nuclear Import of Viral Proteins Lacking NLSs

Although many viral proteins accumulate in the nucleus of a host cell, not all of these proteins contain effective NLSs to direct their final destination. The resulting question is how these viral proteins, which lack NLSs, are transported into the nucleus. One possible explanation is that some small proteins (<40 kDa) can passively diffuse between the cytoplasm and nucleus, and NLSs are not necessary for their nucleocytoplasmic shuttling. Larger proteins (>40 kDa) can be efficiently mediated into the nucleus with the assistance of other proteins. Of course, the possibility that some viral proteins may contain unconventional NLSs to facilitate reaching their final destination in the nucleus is not precluded, and this pathway of nuclear import may be similar to the dynamic trafficking of STAT5, which depends on an unconventional NLS [21].

However, it remains a challenge for scientists to identify the mechanism by which those large viral proteins lacking NLSs are transported into host cell nucleus. Some viral proteins lacking NLSs can enter the nucleus for expression regulation of viral genes and facilitation of viral assembly. The family *Baculoviridae* comprises a large and diverse group of rod-shaped, enveloped, dsDNA viruses, and is divided into four genera: *alpha*, *beta*, *gamma* and *delta*, according to their evolutionary relationship [95]. Over 600 different baculoviruses have been identified and the genomes of 81 have been sequenced. The genomes range in size from 80 to 180 kb, encoding between 90 and 180 genes [96,97]. AcMNPV is the best-studied baculovirus and most extensively used vector for protein expression. Baculovirus replication and nucleocapsid assembly take place in the nuclei of infected cells. For example, the transport of some baculovirus proteins lacking NLSs into the nucleus is mediated by the formation of protein complexes. Prominent examples are the baculovirus proteins P143 and P78/83. The transport of helicase protein P143 into the nuclei of infected cells requires the assistance of the ssDNA binding protein LEF-3 [98,99]. Additionally, the nuclear entry of nucleocapsid protein P78/83 is mediated by the 41.5-kDa viral nucleocapsid protein BV/ODV-C42, containing a putative NLS motif (^357^KRKK) at the C terminus, which potentially functions as a functional NLS in the nuclear import of P78/83 and BV/ODV-C42 complexes [100]. 

The nucleocapsid proteins used for viral assembly need to be transported into the nuclei of infected cells. Proteomics research has revealed that the composition of AcMNPV BV nucleocapsids include P6.9, VP39, VP1054, VLF1, 38K, Ac109, 49K, BV/ODV-EC27, PP78/83, BV/ODV-C42 and VP80 [35]. Ac102 is also part of the AcMNPV BV nucleocapsid [101]. In all known BV capsids, only BV/ODV-C42 and VP80 contain a putative cNLS motif (^357^KRKK and ^424^KRSAEDDLLPTRSSKR, respectively) [102]. To examine whether some potential NLSs exist in the sequences of all the BV nucleocapsid proteins, the online software of cNLS mapper (http://nls-mapper.iab.keio.ac.jp/cgi-bin/NLS_Mapper_form.cgi) has been exploited to predict importin-α-dependent NLSs. cNLS mapper results show that VP39, 38K, BV/ODV-EC27, and VLF-1 contain one potential NLS, whereas two potential NLSs are present in VP80. The potential cNLSs of the BV nucleocapsid proteins are shown in Table 2. However, it remains to be demonstrated whether these sequences are functional NLSs and mediate the nuclear import of baculovirus nucleocapsids. Nevertheless, there are some BV nucleocapsid proteins lacking NLSs in their sequences (Table 2). Except for P78/83, it remains unclear how these nucleocapsid proteins lacking NLSs enter the nuclei of infected cells. It is hypothesized that those proteins lacking NLSs are transported into the nuclei with assistance from other proteins, a model which requires further research for confirmation. 

## 6. Conclusions and Future Studies

It remains a major challenge to develop effective antiviral agents for therapy of some infections. Chronic HBV infection is used as an example to demonstrate the difficulty in the development of antiviral agents. HBV attacks the liver and triggers both acute and chronic disease, causing a major global health burden [103]. However, cure is rarely achieved using current antiviral therapies. Although many antiviral agents against viral DNA polymerase have been developed for the treatment of viral infection, infectious viruses easily rebound in patients and the viral load increases significantly within a short time after use of the antiviral drug is stopped. Chronic HBV infection is a notorious example. Additionally, HBV-specific immunity is an interesting target for new therapeutic strategies. Although promising, the immunotherapy approach is labor intensive, time-consuming and expensive. These setbacks restrict the widespread use of immunotherapy in patients with viral infections. Therefore, there is an urgent need for the development of new therapeutic options, and the development of effective antiviral agents is required to improve long-term quality of life.

DNA viruses are internalized into the cytoplasm by binding to receptors on the host cell surface during infection. Subsequent transport of the viral genome and some proteins into the nucleus is typically mediated by NLSs, facilitating viral replication and assembly in the nucleus of the host cell. Extensive research has revealed the pleiotropic roles of NLSs during the viral life cycle. In the process of viral propagation, NLSs are required for the viral life cycle, especially for the nuclear import of progeny viral proteins. Some viral proteins are involved in viral replication, transcription, gene expression regulation, and assembly, and these proteins must be transported from the cytoplasm to the nucleus to facilitate the production of mature virions. On the other hand, dysfunction of NLSs blocks or impairs the production of infectious virions.

Although sequence diversity of NLSs exists in different proteins, NLSs are able to direct target proteins into the nuclei of infected cells to ensure the production of progeny virions. However, mutations or deletions of NLSs usually make the corresponding proteins accumulate in the cytoplasm, leading to failure of production of progeny virions. Therefore, an alternative therapeutic strategy to control a number of viral infections, such as HBV, may involve blocking nuclear import of some viral proteins. 

## Figures and Tables

**Table 1:** Identification of nuclear localization signals (NLSs) from virally encoded-DNA polymerases.

Num	Virus Name	Viral Genome (size)	dsDNA or ssDNA	Viral Polymerase	NLS Motif	References
1	VACV	192 kb	dsDNA	E9	No NLS	[58]
2	ASFV	189 kb	dsDNA	PolX	No NLS	[25,87]
3	HAd2V	36 kb	dsDNA	Ad2V Pol	RARR^11^, RRRVR^29^, RARRRR^46^	[69]
4	HCMV	236 kb	dsDNA	UL54	NLSA (PAKKRAR^1159^), NLSB (PRRLHL^1227^)	[71]
5	HSV-1	153 kb	dsDNA	UL30	RRMLHR^1229^, PRRSRLW^130^, PAKRPRETPSPADPPGGASKPRK^1136^	[70,72]
6	HBV	3.2 kb	dsDNA	P protein	a bipartite nuclear localization signal (residues K90-K91, K104-R106)	[34]
7	AcMNPV	134 kb	dsDNA	DNApol	DNPGKKRKSTDDNEGPSPKRRVIT^827^, CSVKRKRDDD^948^	[73]
8	SpltNPV	139 kb	dsDNA	DNApol	QE PPA KRARMPT^838^	[74]
9	PRV	143 kb	dsDNA	UL42	KRPAAPRMYTPIAKRPR^370^	[75]
10	BmBDV	VD1 (6543 nts); VD2 (6022 nts).	ssDNA	BmBDV pPolB	Unclear	[51]
11	B19 virus	5.6 nts	ssDNA	No	No	[81,88]
12	HBoV1	5.3 nts	ssDNA	No	No	[79,80]

Abbreviations: VACV vaccinia virus, ASFV African swine fever virus, hAd2V human adenovirus type 2, HCMV human cytomegalovirus, HSV-1 herpes simplex virus type 1, HBV hepatitis B virus, AcMNPV Autographa californica multiple nucleopolyhedrovirus, SpltNPV Spodoptera litura nucleopolyhedrovirus, PRV pseudorabies virus, BmBDV Bombyx mori bidensovirus, HBoV1 human bocavirus 1, dsDNA double-stranded DNA, ssDNA single-stranded DNA.

**Table 2:** Possible classical NLSs of budded virus nucleocapsid proteins.

Num	Target Proteins	Predicted MW	AcMNPV ORF	Homlogs in BmNPV	Potential NLS
1	VP39	39 kDa	89	BmNPV Orf76	^52^HLIKRFKMS
2	38K	38 kDa	98	BmNPV Orf86	^13^RLNDAIIKRHVLVLSEYADLKYLGFEKYKFFEY
3	BV/ODV-EC27	34 kDa	144	BmNPV Orf128	^2^KRIKCNKVRTVTEIVNSDEKIQKTYEL
4	VP80	80 kDa	104	BmNPV Orf92	^424^KRSAEDDLLPTRSSKR; ^464^YEKESKRRKLEDEDF
5	VLF-1	44 kDa	77	BmNPV Orf67	^225^LIKRGKLHSDTINLKRKRSRNN
6	BV/ODV-C42	42 kDa	101	BmNPV Orf 89	^357^KRKK
7	P78/83	61 kDa	9	BmNPV Orf2	No NLS
8	49K	55 kDa	142	BmNPV Orf126	No NLS
9	Ac109	45 kDa	109	BmNPV Orf96	No NLS
10	VP1054	42 kDa	54	BmNPV Orf46	No NLS
11	Ac102	13 kDa	102	BmNPV Orf90	No NLS

Abbreviations: MW, molecular weight, NLS: nuclear localization sequence; ORF: open reading frame; BV: budded virus.
